# Female youth and mental health service providers' perspectives on the JoyPop™ app: a qualitative study

**DOI:** 10.3389/fdgth.2023.1197362

**Published:** 2023-09-27

**Authors:** Ishaq Malik, Arnaldo Perez, Elaine Toombs, Fred Schmidt, Janine V. Olthuis, Jaidyn Charlton, Elizabeth Grassia, Crystal Squier, Kristine Stasiuk, Tina Bobinski, Aislin R. Mushquash

**Affiliations:** ^1^ Department of Psychology, Lakehead University, Thunder Bay, ON, Canada; ^2^ Faculty of Medicine and Dentistry, University of Alberta, Edmonton, AB, Canada; ^3^ Children’s Centre Thunder Bay, Thunder Bay, ON, Canada; ^4^ University of New Brunswick, Fredericton, NB, Canada; ^5^ Dilico Anishinabek Family Care, Fort William First Nation, ON, Canada

**Keywords:** mobile health (mHealth) app, the JoyPop app, qualitative, Technology Acceptance Model (TAM), youth, service providers, mental health, resilience

## Abstract

**Introduction:**

Mobile health (mHealth) apps are a promising adjunct to traditional mental health services, especially in underserviced areas. Developed to foster resilience in youth, the JoyPop™ app has a growing evidence base showing improvement in emotion regulation and mental health symptoms among youth. However, whether this novel technology will be accepted among those using or providing mental health services remains unknown. This study aimed to evaluate the JoyPop™ app's acceptance among (a) a clinical sample of youth and (b) mental health service providers.

**Method:**

A qualitative descriptive approach involving one-on-one semi-structured interviews was conducted. Interviews were guided by the Technology Acceptance Model and were analyzed using a deductive-inductive content analysis approach.

**Results:**

All youth (*n *= 6 females; *M*_age_ = 14.60, range 12–17) found the app easy to learn and use and expressed positive feelings towards using the app. Youth found the app useful because it facilitated accessibility to helpful coping skills (e.g., journaling to express their emotions; breathing exercises to increase calmness) and positive mental health outcomes (e.g., increased relaxation and reduced stress). All service providers (*n *= 7 females; *M*_age_ = 43.75, range 32–60) perceived the app to be useful and easy to use by youth within their services and expressed positive feelings about integrating the app into usual care. Service providers also highlighted various organizational factors affecting the app's acceptance. Youth and service providers raised some concerns about apps in general and provided recommendations to improve the JoyPop™ app.

**Discussion:**

Results support youth and service providers' acceptance of the JoyPop™ app and lend support for it as an adjunctive resource to traditional mental health services for youth with emotion regulation difficulties.

## Introduction

Mental health difficulties are increasing among youth in Canada, and barriers to receiving services (e.g., limited community-based programs, long wait times) are prominent (1). These access barriers are particularly salient in rural and remote areas like Northwestern Ontario, Canada, compared to more populous urban areas (2, 3). Limited access to mental health services and long wait times can profoundly impact youth by increasing social dysfunction, decreasing treatment motivation, prolonging physical and emotional distress, and exacerbating mental health conditions (3, 4). Novel interventions are required to address these challenges.

The Changing Directions, Changing Lives, Mental Health Strategy for Canada, developed by the Mental Health Commission of Canada, has recommended increasing e-mental health (eHealth) interventions to help meet the increasing demand for mental health supports (2). Notably, the Mental Health Commission of Canada reports that youth in Canada are open to using technology for mental health care and that eHealth interventions may benefit Indigenous people and youth in rural and remote areas (5). A recent systematic review of the acceptability and feasibility of eHealth interventions tailored toward the mental health of Indigenous youth found that service users (ages 12–25) and providers (e.g., Elders, frontline workers) report positive attitudes towards eHealth interventions (6).

Mobile health (mHealth) apps (i.e., medical and public health practices delivered via mobile device applications) are one type of eHealth intervention discussed as a possible solution in addressing unmet needs for youth mental health support in Canada (3, 5). App-based solutions have some advantages over traditional services as they provide increased availability and access to support and reduced geographical barriers (7–9). mHealth apps can also be effective as adjuncts to regular services for improving self-management of mental health related difficulties among youth, adolescents, and adults (9–14). However, there are significant gaps between the large number of mHealth apps available and data supporting claims made about their effectiveness (12, 15). Further, the evidence on the effectiveness of mHealth apps designed for youth (12–25) is limited and mixed (11, 14). This was highlighted by a recent systematic review of 11 RCTs (attention controls or wait-listed peers) examining the effectiveness of mHealth apps tailored toward youth [1,706 adolescents and young adults; (11)]. The results showed that four of eight studies on apps targeting depression showed moderate to large improvements, four studies examining overall mental health reported significant small to medium positive effects, three of seven studies found significant and positive effects in reducing anxiety, four studies targeting distress only showed small and non-significant effects, and two studies examining stress showed significant positive effects (11). The authors concluded that more long-term evaluations and studies are needed within diverse clinical, gender, and ethnic youth populations before mental health services can adopt mHealth apps into care (11).

Another consideration related to mHealth app integration is the issue of app engagement. Showing app effectiveness via change in quantitative outcome measures does not guarantee that an app will be used or accepted, and the evidence-based content of apps is often unrelated to their popularity (16). Research has shown that approximately 70% of patients who used or were invited to use mHealth technologies stopped prematurely or declined, with lack of engagement cited as a major contributor (17). Furthermore, the unique needs of service providers (e.g., managers, clinicians) and organizations, which are often highly regulated and have budgetary concerns, can facilitate or prevent app integration into healthcare services (18, 19). Consistent with mHealth app assessment frameworks (20) and Canadian recommendations, future research on developing and evaluating mHealth apps requires a multimethod approach (5, 20). While establishing effectiveness of an app for improving target outcomes is important via quantitative methods, qualitative research is needed to examine user perspectives, identify facilitators and barriers of use, and to reveal specific features, designs, and functions that help with engagement and long-term uptake (21, 22). Examining user perspectives is essential as perceived challenges can slow the uptake of mHealth into services (17, 23).

The Technology Acceptance Model (TAM) is one of the most used frameworks in qualitative studies examining factors influencing users' and service providers’ acceptance of mHealth apps (18, 24). This framework posits that *perceived usefulness* and *perceived ease of use* are strong determinants of attitudes, which in turn influence the *intention to use* technology (24, 25). Extensions are often added to the TAM framework when evaluating app acceptance in complex healthcare settings as these extensions better capture important organizational factors (e.g., available resources and time, consistency with values and needs) (25, 26). For example, the *facilitating conditions* construct from the Unified Theory of Acceptance and Use of Technology [UTAUT; (26)] can account for organizational factors affecting service providers' acceptance of new technology (18, 26, 27). The present study sought to evaluate the acceptance of an mHealth app (JoyPop™) among a clinical sample of youth and service providers in Northwestern Ontario using the TAM framework.

The JoyPop™ app is a resilience-building mHealth app designed for youth (12–25) (28–30) that was conceptualized and developed using resilience theory. Resilience theory is a strength-based approach that focuses on increasing promotive factors rather than reducing youth deficits or risk exposure (31, 32). Promotive factors are social, individual, and environmental factors that can reduce the likelihood that an individual exposed to stress or adversity progresses to pathology (31, 32). Promotive factors are further understood as assets, which occur within an individual (e.g., emotion regulation) and resources, which occur outside an individual [e.g., social connectedness; (32)]. Emotion regulation and social connectedness are recognized as some of the most important facets of resilience (33, 34).

**Figure 1:**
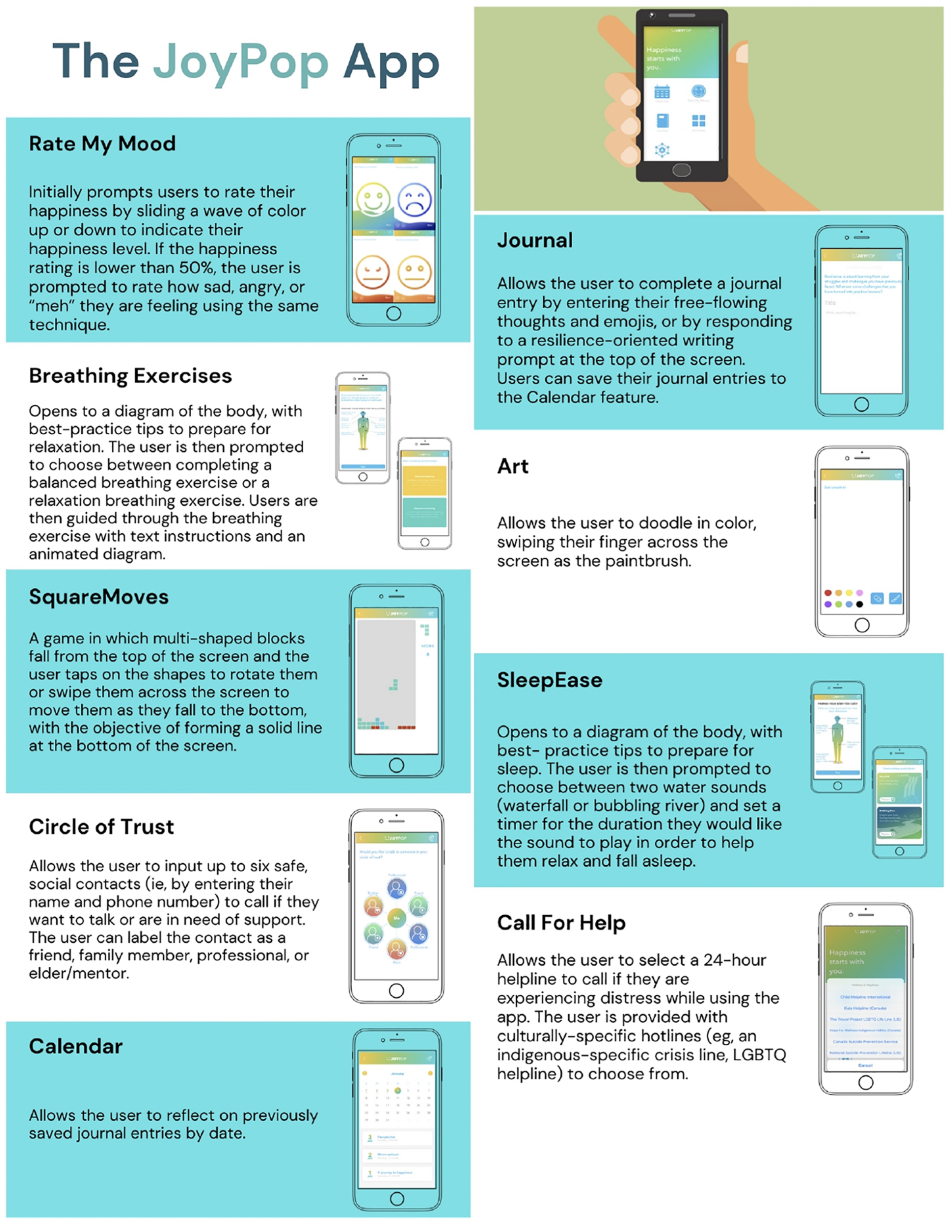
The JoyPop app.

The JoyPop™ app (see Figure 1) focuses primarily on improving promotive assets by helping individuals learn, practice, and implement evidence-based emotion regulation skills that increase self-monitoring and self-awareness (28, 29). More specifically, the Rate My Mood feature helps youth identify, differentiate, and manage emotions (35). The Journal feature provides an opportunity to express thoughts and emotions in words or emojis, and the Calendar provided an opportunity to reflect on past Journal entries (36, 37). Breathing Exercises support relaxation and refocusing (38). A Tetris-like game called SquareMoves provides a helpful distraction (39). The Art feature allows users to express their creativity freely (40). The SleepEase feature provides users with best-practice sleep tips and soothing water sounds to aid sleep onset (41). The app also aims to increase promotive resources by facilitating opportunities for individuals to connect with social and mental health supports. The Circle of Trust feature facilitates quick and easy access to personal social support networks. The Call for Help feature provides instant access to 24-h helplines. When used over time, the improved emotion regulation skills can become habitual responses to stress and improve overall resilience (33, 34, 42). Strength-based mHealth apps, like the JoyPop™ app, can provide youth with 24-h access to evidence-based skills and resources to foster their inherent resilience by up-regulating the positive (e.g., social support), down-regulating the negative (e.g., emotion labelling), and transcending the self [e.g., mindful breathing; (34)]. Moreover, the JoyPop™ app's focus on emotion regulation aligns with recommendations that mHealth apps emphasize the transdiagnostic treatment of mental health conditions (43) and target overall well-being and coping instead of specific mental health disorders (44, 45).

The framework for developing the JoyPop™ app involved a cumulative research and parallel consultation approach. Essential to app development was research showing that improved self-reflection and self-regulation can increase resilience and reduce the association between adversity and adverse mental health outcomes (46, 47). App features were developed based on these findings and consequent consultations by team members, youth, service providers, and clinician-scientists (28). After an initial version of the app was created, revisions of features and designs were assessed and implemented using input from youth involved in child welfare and victim services and providers working with those youth (28).

The JoyPop™ app is particularly relevant in Northwestern Ontario where access to mental health services are especially limited (2, 3) and local data showing that many youth seeking services present with emotion regulation difficulties (48). Moreover, Indigenous youth in this region also show significant challenges related to past trauma and adverse childhood experiences [ACEs; (49)]. This is consistent with evidence from a recent systematic review showing that Indigenous people experience more ACEs (mean score of 2.5–3.05) than non-Indigenous people [mean of 1.36; (50)]. Research has shown that experiencing adversity during childhood is associated with the development of autonomic nervous system alterations that contribute to heightened sensitivity to environmental demands, increased biological and emotional reactivity in response to stress, and less capability for adaptive self-regulation (51, 52). The JoyPop™ app's strength-based approach in fostering resilience and improving emotion regulation skills may help to mitigate the impact of this past trauma and adversity (53, 54).

The JoyPop™ app has a growing multi-method evidence base supporting its use with youth and young adults in Northwestern Ontario. For example, first-year undergraduates at a university in Northwestern Ontario [*N *= 156, 78.8% female, *M*_age_ = 19.02 (*SD *= 2.90), range 16–38, 87.8% were 19 years or younger] used the app for four weeks and showed significant improvements in depressive symptoms and emotion regulation (29). Notably, greater improvements in emotion regulation were evident for those with early adversity and trauma (e.g., abuse, neglect) (29). A follow-up qualitative descriptive study was conducted with 30 undergraduates [80% female, *M*_age_ = 18.77 (*SD *= 2.30), range 16–29, 93% being 19 years or younger] who had used the app over four weeks (30). Participants highlighted important facilitators of use (e.g., increased self-monitoring and expressive opportunities), barriers to use (e.g., lack of variety and editing), positive outcomes of use (e.g., improved self-awareness and emotion regulation), and recommendations for improvement (e.g., adding and enhancing features). This study provided key insight into features of the app that were useful and engaging to users, along with recommendations to continue improving user experience while promoting long-term engagement (30). While promising, initial evaluations of the JoyPop™ app were limited to older youth within a university setting (29), and qualitative feedback was gathered without a guiding theoretical framework (30). Moreover, it is unknown how service providers felt about the app and its use or relevance for the clients presenting for mental health services.

To better understand the relevance and utility of the JoyPop™ app for youth seeking mental health services in Northwestern Ontario, this study evaluated the acceptance of the JoyPop™ app among a clinical sample of youth and service providers at the two largest mental health agencies in Northwestern Ontario. Specifically, we evaluated (1) youth acceptance of the JoyPop™ app using the TAM (i.e., perceived usefulness, perceived ease of use, and attitudes towards use); and (2) service providers' acceptance of the JoyPop™ app in a mental health setting using the TAM plus the facilitating conditions construct from the UTAUT (26). In line with the TAM, we defined acceptance as youth and service providers' behavioural intention to use the app based on perceived usefulness, perceived ease of use, and overall attitudes towards use. With the lack of a consensus and clear definitions of acceptability, acceptance, and adoption in the mHealth literature (55), this study used recommendations suggested by the Technology Acceptance Lifecycle guidelines to improve the quality of communication in technology use research. These guidelines recommend using the term acceptance when a technology is in the initial use phase and when perceptions are being assessed after using a technology (55).

## Materials and methods

### Design

The present study was part of a larger pilot study of the JoyPop™ app. For the pilot study, youth 12–18 years old who were seeking or receiving mental health services from *[blinded for peer review]* were provided with the app and asked to use it across four weeks. Youth completed outcome assessments at baseline, after two weeks, and after four weeks. The pilot study included 41 youth (63.4% female; 70.4% Indigenous, 24.4% White) with a mean age of 15.0 (*SD* = 1.41). Twenty participants who completed all outcome assessments during the pilot study were invited to participate in the present qualitative study. Service providers affiliated with youth services from pilot study sites were invited to participate as well. This qualitative study was guided by qualitative description, which is best suited when a straightforward description of a phenomena is desired (56). Conceptually, the TAM and UTAUT informed the study design, especially data collection and analysis. The present study was reviewed and approved by [*blinded University's*] Research Ethics Board and the ethics committee/advisory boards of *[blinded for peer review; partner mental health agencies]*.

### Participant sampling and recruitment

#### Youth

All youth were recruited using purposeful sampling, which identifies and selects individuals with rich knowledge or experience related to the primary purpose of a research study (57). Consequently, we sought feedback and invited youth with significant experience using the JoyPop™ app to obtain comprehensive feedback on its acceptance. Youth were contacted via email, asking if they were interested in participating in the qualitative study following their completion of the pilot study.

#### Service providers

We used purposeful sampling to identify service providers who were informed about the JoyPop™ app and interested in participating. Service providers at both pilot study sites were initially informed about the JoyPop™ app during the launch and promotion of the pilot study (e.g., through presentations and weekly emails from the research team). Some service providers at the sites had referred youth to the pilot study whereas others had less direct involvement with the pilot study. We also used snowball sampling by asking participating service providers if they knew other service providers that may be interested in the study (57).

### Data collection

Youth interested in participating were provided general information about the study and contacted to schedule a time and location to complete the interview. Youth provided informed consent prior to participating. Service providers interested in participating communicated with the research team to arrange a date for the interview and were sent an additional information email, which included a consent form, PowerPoint document and video describing the rationale and function of each app feature, a link to the JoyPop™ website and eBook, and multiple videos describing the app. Before each interview began, service providers had the option of viewing a 4-min JoyPop™ feature review video if they wanted a reminder about the design and features (58).

The first, second, and last author created the youth interview guide in consultation with partner mental health agencies and based on the TAM. The guide included questions such as “How did using the app affect your mental health, if at all?” and “What would make the app more useful to you?” The first, second, and last author developed the service provider interview guide in consultation with partner mental health agencies and based on the TAM and the facilitating conditions construct of the UTAUT. The service provider interview included questions such as “What is your impression of the usefulness of the app as an adjunct to usual services for youth in and/or seeking mental health care?” and “What factors in your organization would make integrating this app into usual care easier or more difficult?” The first author pilot tested the interview guides (see Supplementary Material S1) with two youth and two service providers. The first author reviewed the pilot interview audio recordings and transcripts with the last author and discussed participant experiences during these interviews with the second and last author. Interview guides were deemed adequate upon review and discussion because they were understood clearly, facilitated participant comfort to provide feedback in a nonbiased manner, and elicited appropriate information related to the TAM constructs of interest and study purpose. The first author conducted all interviews between December 2021 to March 2022. Interviews were audio recorded, anonymized, and transcribed verbatim. Interviews were conducted virtually (i.e., via Zoom), by phone, or in person based on participant preference. All participants received $10 CAD upon completing the interview.

### Data analysis

The first, second, and last authors were the only research team members involved in data analysis. The first author checked transcripts for accuracy before data analysis, imported them into NVivo Software 12, and coded all the data. We used deductive-inductive content analysis to analyze youth and service provider data to provide a condensed and thorough description of app acceptance in the form of a conceptual map (59). This approach consists of three main phases: preparation, organizing, and reporting (59). We used constructs from the TAM and UTAUT to provide the organizing categories during data analysis.

In the *preparation* phase, we selected individual interviews as the unit of analysis. The first author also immersed himself in the data by transcribing each interview and reading through each transcript several times (59, 60). In the *organizing phase*, the first author coded the relevant data line-by-line into organizing categories (constructs) of the chosen frameworks. The first author inductively coded the data and grouped it into generic categories and sub-categories within the organizing categories. Additional organizing categories were inductively developed to create generic categories, and sub-categories to account for the data that did not fit into the constructs suggested by the TAM and UTAUT. Participants' terms were used to name the developed categories and sub-categories. Finally, in the *reporting phase*, two conceptual maps were developed to summarize the analysis of the youth and service provider data. The first author performed all the steps of the analysis, including coding, categorization, and mapping, and discussed all steps with the second and last authors who have extensive experience in qualitative research. We resolved discrepancies throughout the analysis process by consensus.

Data saturation was based on category saturation and the richness (quality) and thickness (quantity) of the data (61). Interview guides were structured to elicit information relevant to app acceptance and pre-determined theoretical categories to help achieve data saturation (61, 62), and as a result of the small sample size of the population (i.e., youth with significant experience using the app, service providers with sufficient knowledge of the app) and ethical concern of reducing participant burden, data saturation was assessed after four interviews from each group. The first author then discussed the commonalities, quality, and quantity of data emerging in each generic and sub-category nested within relevant pre-determined main categories with the second author. Because of the common data from youth and service providers, it was determined by consensus that two more interviews would be conducted to assess if saturation had been reached. After two more interviews, it was determined that category saturation was reached (i.e., interviews and analytic procedures provided no new material for analysis).^1^

### Reflexivity and trustworthiness

We completed and used the Consolidated Criteria for Reporting Qualitative Research (COREQ; see Supplementary Table S2) as a guide to create a detailed and comprehensive report of the research study (63). To ensure trustworthiness throughout the data collection and analysis processes, we applied various strategies (i.e., credibility, dependability, confirmability, transferability) outlined by Elo (64) and Lincoln (65). We have highlighted specific strategies and methods used to establish trustworthiness throughout data collection and analysis in Table 1.

**Table 1:** Trustworthiness strategies used throughout data collection and analysis.

Strategy	Method
Credibility	• Established via first author having past interviewing experience, proper training, and rigorous preparation (65). The first author was trained in essential interviewing techniques (e.g., proper use of prompts, neutrality) and supervised (e.g., reviewing initial transcripts and providing feedback) throughout the study by the fourth and last author, who have extensive clinical and research interviewing experience (65). • Interview questions were enhanced by the team assessing their appropriateness for obtaining data relevant to the research questions and guiding frameworks (64). The team reviewed and validated questions to ensure they were presented in a neutral and open manner to avoid indications of the researchers’ opinions (65). • Interviews were pilot tested with youth and service providers to test that the questions were suitable for the research question before being applied to the entire sample (65). • Member checking was used throughout interviews by using pauses, direct questioning, clarification, and reflective listening to improve the credibility and accuracy of the information recorded (65). • Throughout data analysis, the first, fourth, and last author engaged in an iterative, constant comparative method to discuss the coding procedures, findings, content within categories, and hierarchy of categories (65). At each stage of data analysis (i.e., preparation, organization, reporting), the first, fourth, and last author engaged in discussions to resolve differences, reach a consensus, and improve the reliability of findings (64, 65).
Dependability	• IM practiced interviews and wrote in reflexive diary before and after each interview to assess power dynamics, difficulties, and differences they may have experienced to improve self-awareness, neutrality, and dependability of the collected data throughout interviews (65, 66). • Tracking the important contextual details (e.g., interview location and presence of others) throughout interviews in the reflexive journal (67). • Use of member-checking during interviews (e.g., pauses, reflective listening, clarification) and a detailed audit trail to track all decisions (65, 68).
Confirmability	• Pretesting the categorization matrix in a pilot phase (65, 69) and having the research team review and reach agreement with the procedure and analysis while providing feedback to improve the process (64, 65). • Enhanced by using illustrative quotes from participants to ensure data accurately represented the participants’ view and not the researchers’ biases or perspectives (56, 59).
Transferability	• Detailed audit trail tracking all analysis procedures, decisions, ideas, and discussions. Detailed analytical memos kept throughout so the research team could reflect on their decision-making processes and make adjustments throughout This procedure can also help other researchers follow the analytical decision trail (65, 66) • Use of validated theoretical constructs to improve transferability of the results by improving the organization and focus of study, clarifying the meaning of the research, and facilitating the identification of the strengths and weaknesses of the study (70).

## Results

### Youth

#### Participant demographics

Seven youth, out of the 20 invited, agreed to participate in the study. One youth chose to stop participating after the first two interview questions due to discomfort engaging in a one-on-one interview. This resulted in a final sample of 6 female youth with a mean age of 14.60 (*SD = *1.63; range 12–17 years). Three participants self-identified as White, and three self-identified as Indigenous. Five participants were in high school, and one was in elementary school. The 6 youth that participated were similar in age, gender, and ethnicity to those that did not complete an interview or choose to participate [*N* = 14, *M*_age _= 15.0 (*SD *= 1.84), range 12–18, 85.7% female, 64.3% Indigenous, 35.7% White]. Interviews averaged 26 min in length (*SD = *9.31; range 10–42 min) and occurred virtually over Zoom (*n *= 4), by phone (*n *= 1), and in-person (*n *= 1).

### Youth acceptance of the JoyPop™ app

**Figure 2:**
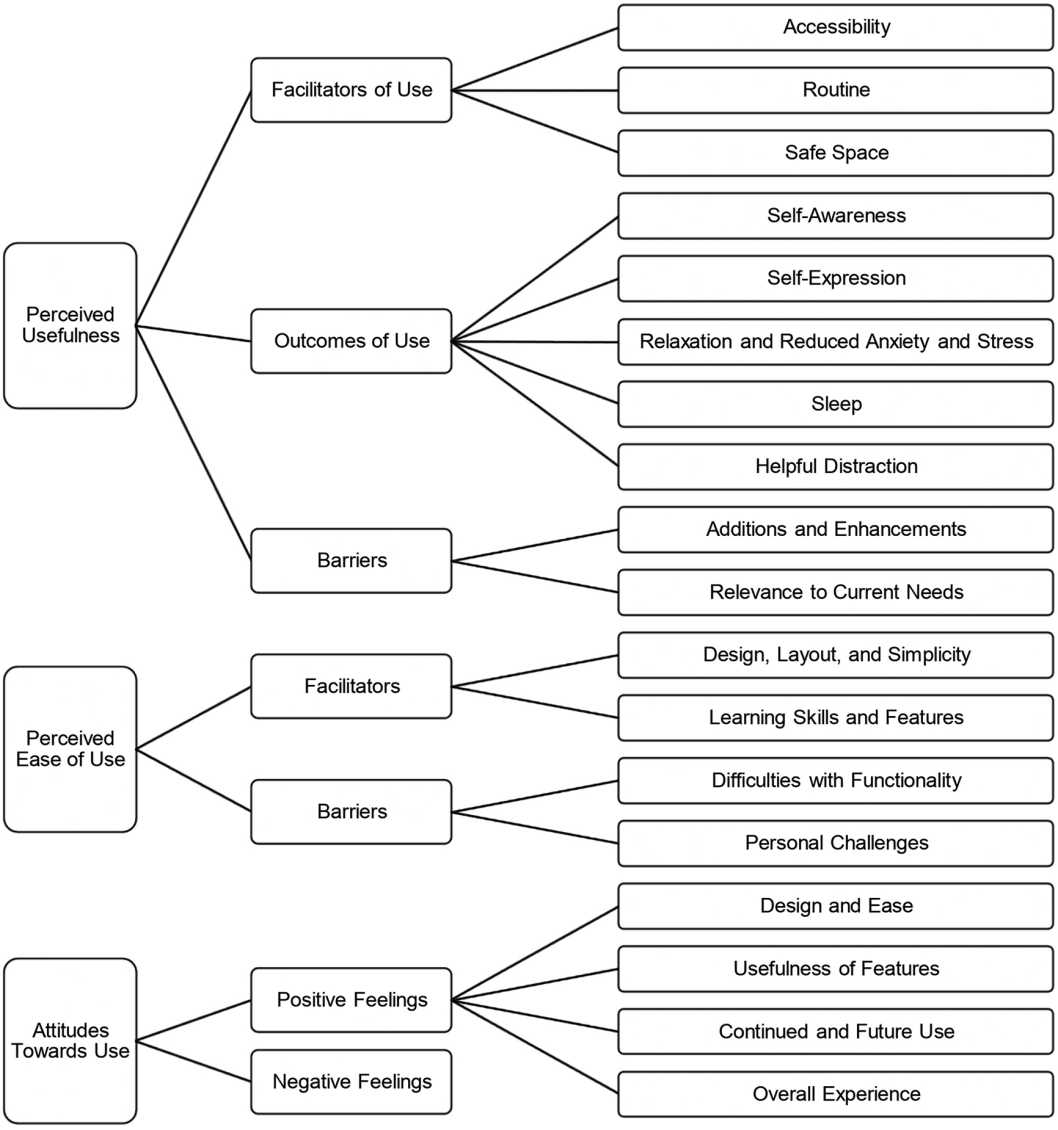
Conceptual map summarizing youth acceptance of the JoyPop app using the technology acceptance model. The technology acceptance model includes the constructs of perceived usefulness, perceived ease of use, and attitudes towards use. Organizing categories (left), generic categories (middle), and sub-categories (right).

The conceptual map (with several generic categories and sub-categories under each organizing category) drawn from the deductive-inductive analysis is presented in Figure 2. We describe each organizing category and its associated generic categories and *sub-categories* below. Quote numbers (Q#) have been provided within the text to guide readers to specific quotes in Table 2 that illustrate participant experiences and the sub-categories.

**Table 2:** Categories and illustrative quotes summarizing youth acceptance of the JoyPop™ app.

Organizing category	Generic category Sub-category	Quote number (Q#), quote, participant number (P#)
Perceived usefulness	**Facilitators of use**	
	Accessibility	**(Q1)** “It's just kind of something that you can use whenever you feel like you need it or you feel upset or you just want to distract yourself if your just bored or if you want to go to sleep, you just use the app”. [P3] **(Q2)** “If they [my counsellor] cancelled on me or something like that and I needed to talk to somebody or talk to something right away and I just needed to vent to something, but nobody was there; I could use the app as a kind of relief for the time being”. [P5] **(Q3)** “…right there in the moment if you're feeling like you need it, so many things [crisis lines] pop up when you hit that”. [P2]
	Routine	**(Q4)** “It was just kind of something that just kind of became part of my daily routine I guess and…it was like, oh ok sweet, I feel kind of crappy right now, I am going to go on this right now and like calm down”. [P3] **(Q5)** “I feel like it gave me some type of structured routine. More so because when I started it [using the app], I was kind of getting back into everything [day-to-day life]”. [P2]
	Safe space	**(Q6)** “I don't talk to many people about what's going on in my head and if I do then I feel like I'm like a crazy person or something so it's nice to just have one space to write without feeling like I am going to get judged for it”. [P1] **(Q7)** “[The journal entry] is on a place other than notes because I used to journal in notes but then people become ever nosey on people's phones. So, I don't like to use my notes anymore and like this [the app] is just like my [own] space”. [P2]
–	**Outcomes of use**	
	Self-awareness	**(Q8)** “I often have emotions like that where I don't know where they're coming from. And even just to look back on that [journal entries] and be like ‘oh, maybe this is why I have been in this type of mood for the past two days because this is what happened this day’, and even like that”. [P2] **(Q9)** “The one with your feelings helped you with your mood if you're feeling down you just go, ‘oh, I'm just going to play this’ or if it's like down, ‘oh I should just do this more’”. [P3]
	Self-expression	**(Q10)** “You can just go on [the app] and go off on the journal and just say whatever”. [P3] **(Q11)** “Well, it sort of helps especially when I get to vent. It helped when I got to vent at the end of the day especially or even in like the beginning of the day because sometimes, I would do the journaling in the morning and the evenings”. [P5]
	Relaxation and reduced anxiety and stress	**(Q12)** “I would use the breathing when like obviously when I would be stressed out, but even like in a good mood just kind of um…like an end of the day type thing like a meditation almost. I would just sit down and just look at it, and breath with it”. [P5] **(Q13)** “Because like usually when I feel sad or something I'll like keep it bottled up inside and there's like…but like when I was able to write it down [using the Journal Feature] it took a lot of pressure off”. [P6]
	Sleep	**(Q14)** “I read all the…all the labelling things [sleep tips] to relax my body and that bit, and it helped. I was having more deep sleeps I found. Like getting in that mindset before bed and like it just being bed kind of thing. Getting my…not only like me ready, but like my mind and body ready for bed, it helped”. [P2] **(Q15)** “It [sleep sound] always reminded me of the sound of rain so it would make me pass out right away”. [P3]
	Helpful distraction	**(Q16)** “Let's just say I got into like an argument with my friends like I did a few times, I would just go on there [the app] and distract myself with the art or starting journaling”. [P3] **(Q17)** “If you're in counselling and you're a bit nervous you could quickly play a game [to distract yourself]”. [P4]
–	**Barriers**	
	Additions and enhancements	**(Q18)** “I think it would be better to use if there's also a place where you can text people like the Kids Help Phone or something. Like if there were like numbers you could text instead of call, and it would bring you to the crisis line. [P6] **(Q19)** “The one thing I would recommend is not only giving it to iOS phones but to also Android phones because that would broaden the spectrum”. [P5]
	Relevance to current needs	**(Q20)** “I didn't really use the sleep features as much because I just…I myself don't have a problem with sleeping so I didn't really need that”. [P5] **(Q21)** “I have a calendar in my phone, so I just use that one and then having two calendars didn't make sense to me”. [P1]
Perceived ease of use	**Facilitators**	
	Design, layout, and simplicity	**(Q22)** “Like everything was right in the home screen area. It's very simple and it's not complicated like trying to setup different apps that I have”. [P2] **(Q23)** “I think it was easy to use because like it's not like it's really complicated. Everything is really simple, so it was just easy to get the hang of it and know what to do”. [P6]
	Learning skills and features	**(Q24)** “[Insight into a past emotion would occur] usually after I looked back. Things take me like a little bit to process everything. So, I am like the type of person that likes to let things sit for a little bit and then go back to them, and with this app it's easy just to go back [to journal entries] because it's on your phone and there”. [P2] **(Q25)** “It's controlled [the Breathing Feature exercises], so there's actually somebody there with a calm voice telling you what to do and that is something that is a lot easier than just doing it by yourself when you're panicking”. [P5]
	**Barriers**	
	Difficulties with functionality	**(Q26)** “[Using the SquareMoves feature] whenever I tried to bring the pieces down it would automatically go to the bottom, so I never knew where it was exactly landing”. [P3] **(Q27)** “With the Art like sometimes you'd press a colour, but it won't choose that colour, and it will say you didn't click it. So, I would go like press it and like colour it and I would ruin my entire drawing because it didn't like choose the colour that I wanted”. [P6]
	Personal challenges	**(Q28)** “I have these little like episodes and they're like really similar to panic attacks and I struggle to breath when I'm having them and then when I try and breathe properly my body gets overwhelmed because it's almost like I forget how to breathe. So, trying to use the breathing exercises was hard but sometimes I was successful in getting to breath properly”. [P1] **(Q29)** “[When using the SquareMoves feature] I would accidentally click without realizing that I clicked because my hand does this thing where it just jolts. So, for me it was more difficult to use because of that issue that I have”. [P5]
Attitudes towards use	**Positive feelings**	
	Design and ease	**(Q30)** “Even like the colours they used [in the app] are nice and calming I found. Like, just like even the way the app is. I like it”. [P2] **(Q31)** “It had nice calming colours even. The home…like the little button that you click on is a smiley face and I love it”. [P5] **(Q32)** “I really liked the journaling one because it would ask you questions, and I would be like oh sweet I'll just go off about this and write a little essay on it”. [P3]
	Usefulness of features	**(Q33)** “Even if you're not calling anyone [in the Circle of Trust feature], just to look on that and see how many people are actually there for you and you know, that is like a really good thing”. [P2] **(Q34)** “Like it helped me…like the entire app helped me so I don't go into a state of panic or like so that I can have like someone to talk to. Like it helped me understand my feelings better, so I don't feel frustrated or like as angry or as panicked”. [P6]
	Continued and future use	**(Q35)** “It was just a cool thing to have and that I am going to keep using and even for my friends that do have Android, like I have shown them the app by screensharing it on my facetime just so they can see… “this would be something you should really get” because I know a lot of my friends they struggle with mental health”. [P3] **(Q36)** “Oh, I think it is a great thing to suggest to people because…I've been suggesting it to a lot of people actually, even my mom. Me and her were talking about it and she was thinking about getting my younger sister it even to help her out because she loves her iPad”. [Participant 2]
	Overall experience	**(Q37)** “My overall experience? It was really good. I found it was a positive thing in my life and not stressful”. [P2] **(Q38)** “It's the first thing that's ever worked for me so I'm definitely happy with it”. [P3]
	**Negative feelings**	
	No subcategories	**(Q39)** “I couldn't understand how to play it [SquareMoves feature] and it was like frustrating”. [P1] **(Q40)** “I kind of felt a little underwhelmed because they were telling me there were going to be games and I expected more than just one game. So, I was a little underwhelmed to see there was only Tetris”. [P6] **(Q41)** “It's only available on Apple, which kind of sucked but it's ok”. [P5]

#### Perceived usefulness

Overall, all participants described helpful facilitators that promoted use of the JoyPop™ app and beneficial mental health outcomes following use, along with barriers preventing the perceived usefulness of the app.

##### Facilitators of use

All participants described how the *accessibility* of the app was essential in facilitating their continued use. Participants primarily described how the app was always available to provide quick, efficient, and timely support when needed, such as when they experienced mental health difficulties, had no one to talk to, or were bored (Q1). Some participants specifically discussed how the app could provide needed support between counselling sessions (Q2). Others talked about how the app simplified access to relevant crisis services via the Call for Help feature (Q3). Half the participants described how the app helped them develop a useful *routine*. For example, some participants began using coping skills regularly when experiencing stress or difficult moods; others talked about the app facilitating the development of a structured routine in their daily lives (Q4 + 5). Four participants discussed how the app was helpful because it provided a *safe space* to work on their mental health. The safe space was discussed in two ways: to have their own space and time for themselves or to work on their mental health privately without judgment (Q6 + 7).

##### Outcomes of use

Four participants discussed how using the app and specific features, such as the Journal, Rate My Mood, and Calendar features, helped them increase their *self-awareness* by improving their abilities to identify, understand, and reflect on their emotions and thoughts. For example, participants discussed how they would reflect on past journal entries using the Calendar feature to understand the antecedents of their feelings or how they would use the Rate My Mood feature to help them understand their emotions and implement coping skills to manage them (Q8 + 9). All participants described how they used the app to foster *self-expression* of their emotions and thoughts by using either the Art feature to visually express themselves or the Journal feature to vent and expressively write about their days (Q10 + 11). All participants discussed how the features of the app (particularly the Journal and Breathing) helped them increase *relaxation and reduce anxiety and stress* (Q12 + 13). Three participants talked about how the SleepEase feature helped improve their overall *sleep*. Sleep quality and onset were improved by integrating the sleep tips and using the sleep sounds (Q14 + 15). Three participants mentioned how the Art, Journaling, and SquareMoves features provided a *helpful distraction* in stressful situations or how they could foresee the features being used as a helpful distraction in situations (e.g., interpersonal, educational) that might result in stress and anxiety (Q16 + 17).

##### Barriers

All youth participants discussed *additions and enhancements* to improve the app's usefulness. Suggested additions included having more features (e.g., educational games and coping skills), adding helpful evidence-based daily tips in the form of notifications (e.g., reminders to exercise), and adding text options for crisis lines (Q18). Participants also recommended enhancements to the app that might make it more useful, such as adding more pencil sizes to the Art feature, increasing the number of emotions in the Rate My Mood feature, and expanding the accessibility of the app across mobile platforms (Q19).

Each participant described personal and contextual factors that reduced the utility of specific features because of their *relevance to their current needs*. These perceptions were not critiquing the usefulness of the features to others who may benefit but were descriptions of why a specific feature was not helpful for their specific mental health needs (Q20) or that they already had other tools that helped fulfill a feature's function (Q21).

#### Perceived ease of use

Youth participants described aspects of the app that either facilitated the ease of learning and/or using the app or barriers that made the app more difficult to learn and/or use.

##### Facilitators

Five participants commented on the app's overall *design, layout and simplicity*, and how it facilitated their adjustment to using the JoyPop™ app. Participants specifically discussed the ease of navigating the app and locating various features. They highlighted how the app's aesthetics (e.g., colour scheme), layout and simplicity made it convenient to use, that everything was organized in one area, and that nothing in the layout was ambiguous. Some participants also found the app easier to use than previous mHealth apps they had tried (Q22 + 23). Five participants mentioned that it was easy to *learn skills and features* within the app and implement them to their benefit, such as using and combining the Journal and Calendar features to reflect on and gain insight into past experiences (Q24). Others discussed how it was easy to use breathing exercises to manage experiences of stress and panic because the feature provided instructions and guidance (Q25).

##### Barriers

Three participants noted difficulties with functionality due to technical issues within features that made them challenging to use. These issues were related to an error when saving moods while using the Rate My Mood feature and design complications in the SquareMoves and Art features (e.g., no undo button; Q26 + 27). All participants discussed personal challenges associated with their mental health, psychomotor difficulties, and personal situations (e.g., time constraints) that made the app difficult to use (Q28 + 29).

#### Attitudes towards use

##### Positive feelings

Four participants expressed positive feelings about the design and ease of learning and using the JoyPop™ app. Many participants found the colours and design of the app aesthetically appealing (Q30 + 31). Some participants expressed positive feelings towards the ease of using specific features because of their built-in supports (e.g., journal prompts) and the simplicity of using them during times of stress (Q32). All participants communicated positive feelings toward the usefulness of features in improving their mental health. For example, some participants found the Circle of Trust feature helpful because it facilitated quick access to social support and/or was a helpful reminder of their social support network (Q33). Participants also discussed how much they enjoyed the SleepEase feature because it helped them resolve their sleep issues, the Journal feature because it helped them reflect on positive experiences and mitigate negative feelings, and the Rate My Mood feature because it increased their insight into their emotions (Q34). All participants expressed positive feelings about continued and future use of the app. Many talked about how they felt good about using the app during the study and would continue using it because of the mental health benefits they acquired. Some participants also discussed how they would recommend the app to friends and family members with mental health difficulties (Q35 + 36). Each participant expressed positive feelings about their overall experience with the JoyPop™ app (Q37). One participant compared their overall experience to previous efforts to improve their mental health (Q38).

##### Negative feelings

Although no subcategories emerged under this generic category and most feelings expressed towards the JoyPop™ app were positive, four participants expressed *negative feelings* primarily directed to the app's lack of variety and the SquareMoves feature because the design made it challenging to guide blocks down (Q39 + 40). Two participants expressed disappointment that the app was only available on iOS devices, preventing them from using it on their personal phones throughout the pilot study (Q41).

#### Supplementary analysis

To compare youth that participated and completed an interview in this qualitative study (*N* = 6) to invited youth that did not complete the interview (*N* = 14), we examined scores on the satisfaction and acceptability construct of the User Experience Questionnaire for Internet-based Interventions [UEQII; (71)], where higher scores indicate greater satisfaction and acceptability (maximum score = 32) that was included for all participants in the pilot study. An unequal independent samples *t-test* showed that scores for the six participants who completed the qualitative study (*M *= 25.5; *SD *= 2.83) did not significantly differ from the 14 that did not complete an interview (*M* = 25.0; *SD *= 0.66).

### Service providers

#### Participant demographics

Seven female service providers (*M_age _= *43.75, *SD = *8.93, range 32–60) participated in the study. All participants self-reported their ethnicity as White. Positions held included managers, counsellors, and program directors. Most participants served clients between the ages of 6–18, with four primarily providing services to Indigenous clients. Participants had an average of 16.25 years of experience working in mental health services (*SD = *8.23). Three service providers were providing counselling for clients who participated in the pilot study, and one was providing counselling for a client who completed an interview. Interviews averaged 32 min in length (*SD = *3.08, range 27–48 min), and occurred virtually over Zoom (*n *= 6) and by phone (*n *= 1).

### Service provider acceptance of the JoyPop™ app

**Figure 3:**
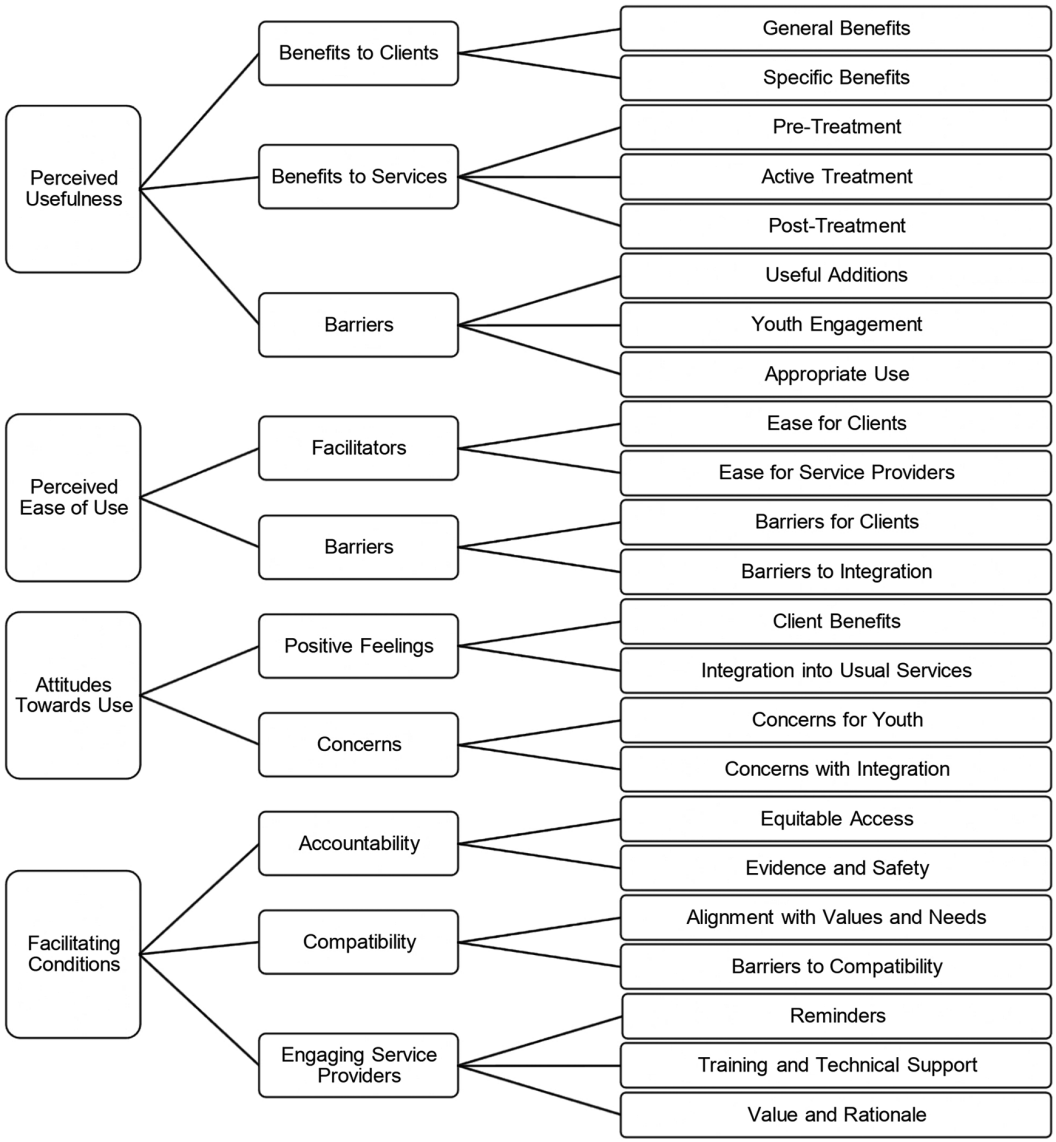
Conceptual map summarizing service providers’ acceptance of the JoyPop app using an extended model of the technology acceptance model. The extended model of the technology acceptance model includes constructs of perceived usefulness, perceived ease of use, attitudes towards use, and facilitating conditions. Organizing categories (left), generic categories (middle), and sub-categories (right).

The conceptual map (with several generic categories and sub-categories under each organizing category) drawn from the deductive-inductive analysis is presented in Figure 3. We describe each organizing category and its associated generic categories and *sub-categories* below. See quote numbers within the text which specify associated quotes in Table 3.

**Table 3:** Categories and illustrative quotes summarizing service providers acceptance of the JoyPop™ app.

Organizing category	Generic category Sub-category	Quote number (Q#), quote, participant number (P#)
Perceived usefulness	**Benefits to clients**	
	General benefits	**(Q1**) “So, just having this extra tool is it gives more access to service and to underserved populations”. [P1] **(Q2)** “It puts your wellness in your own hands and gives you a tool to support you and teach you new strategies”. [P3] **(Q3)** “It could help build habits for using relaxation and coping skills”. [P4]
	Specific benefits	**(Q4)** “It's not just about…you know like rating your mood, it has other like self-care stuff like helping with sleep and breathing exercises and some distraction stuff and journaling, calendar, so it has a lot of other features that can help”. [P2] **(Q5)** “[The app can provide] strategies to cope with some of the negative feelings that you might be having. And also having some proactive strategies, like, this will help you sleep, which is going to help your mental health the next day”. [P3] **(Q6)** “I found the JoyPop app, like that emotional regulation piece was really able to help when they [clients] are escalated, whether it's breathing exercises or the distraction techniques”. [P7]
–	**Benefits to services**	
	Pre-treatment	**(Q7)** “I am hoping that it will help decrease waitlists if some of our youth come, their struggling but maybe they're not as high-risk or have as intense of needs, this app might be just the tool that they need and they may not need counselling”. [P1] **(Q8)** “a lot of them [youth] don't actually want to go and talk to someone about it because they are quite comfortable in their avoidance. Perhaps this [the app] could be like a first step to seeking some help”. [P4]
	Active treatment	**(Q9)** “It's definitely like a supplement to like remind clients of the skills in between sessions”. [P5] **(Q10)** “It could also provide, if clients are using it, it could provide some valuable information for the in-session work in terms of like tracking or understanding moods”. [P4] **(Q11)** “This app just enhances and gives us more tools in the toolbox or more opportunities to provide people that we're trying to help with tools for them to do the work they need to do”. [P6]
	Post-treatment	**(Q12)** “I think when we are transitioning out of counselling, this is a good tool to still provide like some service support after counselling is completed so it's not just a clear cut-off, it's a nice transition piece”. [P1] **(Q13)** “Long-term as a warm hand-off as clients are leaving their service that feel like that they're not leaving with recommendations on paper. But there's actually something they can continue to do that has started in counselling and that continues after counselling”. [P7].
–	**Barriers**	** **
	Useful additions	**(Q14)** “Having other language options. So, some of the youth in our, I'm just thinking of our youth in more northern communities, English may not be their first language or literacy might not be…I think that those considerations need to be considered in the app and that would maybe promote more use with some of our higher risk kids”. [P1] **(Q15)** “I'm not sure what notifications come up but if something came up to like spark that reminder that might be helpful for less motivated youth”. [P5]
	Youth engagement	**(Q16)** “It is a real hit or miss on whether or not they'll buy into it. I have talked to a couple of clients about it, and some have said yes, some have said no, and some don't follow through when they're reached out to. I think if they gave it a chance, they would like it but they're just not following through or they're too cool for it”. [P2] **(Q17)** “I mean like when you're a teenager you don't really want to be like lectured or told what to do and…not that the app does that from like a judgemental perspective but like to…to want to use a coping skill is a hard buy-in for youth”. [P5]
	Appropriate use	**(Q18)** “You have to be really thoughtful about where in the disease progression, and I put mental health in that, where somebody is at. It's getting the right treatment at the right times so if someone is really sick, this probably wouldn't be the intervention I would promote at that point”. [P6]
Perceived ease of use	**Facilitators**	
	Ease for clients	**(Q19)** “It is user-friendly and they're able to like follow through, understand it, and navigate it”. [P7] **(Q20)** “Youth are comfortable with technology so I think that they would be very much confident and comfortable using this”. [P1]”
	Ease for service providers	**(Q21)** “I don't think [it would be hard to integrate]. I mean, like I said we use the [another] app and that implementation was fine. There wasn't, there weren't issues with it so if this is something that we wanted to do then I think that we could do it”. [P3] **(Q22)** “Like as far as I know it's not really that hard to set [clients] up with it like if they download the app and we could talk about it in session, that would be the first session to get them going, it would be pretty easy, I don't foresee that being overly complicated”. [P2]
–	**Barriers**	
	Barriers for clients	**(Q23)** “I think [a barrier is] like cost and then like if youth have access to…an app or like a device they feel comfortable using it on”. [P4]
	Barriers to integration	**(Q24)** “So, then [service providers are] unlikely to recommend the use of an app when they're like ‘I think technology is terrible’ or something like that right? There's personal feelings about the use of an app, personal feelings about the privacy, and um…yeah just like individual comfort with apps or using technology for mental health. Those would be factors”. [P4] **(Q25)** “I think that [service providers are] just busy and it's just an extra…sometimes staff don't always see that putting in the work at the beginning saves you work in the end”. [P1]
Attitudes towards use	**Positive feelings**	
	Client benefits	**(Q26)** “I think it's pretty awesome. Like it's really…it covers a lot. Like it checks off a lot of areas for youth, and there's a lot of really good content”. [P3] **(Q27)** “The positive feelings are that it's accessible and it's confidential”. [P7] **(Q28)** “I mean so far it seems like it is a good app. Like the client that I know that used it said she really liked it”. [P2]
	Integration into usual services	**(Q29)** “There's nothing that would stop me from wanting to integrate it. I think it's good and worthwhile”. [P2] (**Q30)** “I think it's an opportunity to work with kids in different ways and…whether it's pre-treatment, ‘here try this’, or it's after treatment or during treatment…I think its just a great…these kinds of things are great tools”. [P6]
	**Concerns**	**(Q31)** “We also know that a lot of the youth that we service within our agency struggle with reading…the app might seem like more…might be confusing for them because they don't understand the language”. [P1]
	Concerns for youth	**(Q32)** “Like I worry in general about youth like spending so much time in a virtual space. […] So, it's one more app and one more way I think youth are spending more and more time looking at a screen, and sort of existing in a virtual world”. [P4] **(Q33)** “I am just unsure at the success that our clientele would be at following through with the app. So, we could give them the app, but I am unsure at how much would truly follow through especially without an incentive to do it”. [P7]
	Concerns with integration	**(Q34)** “I had concerns around like who owns the information. Because even though within the study there obviously were numbers associated and like not identifying information, there's still the entering of private information into an app that then…when the study's over like who owns that”. [P4]
Facilitating conditions	**Accountability**	
	Equitable access	**(Q35)** “The last question I would have is from an equity perspective, is this app biased to any particular, which they generally are…tend to be white middle class people. How does this adapt for people who aren't in the dominant group?” [P6] **(Q36)** “Just the lack of devices and lack of internet is definitely a struggle [for clients] and as an agency we just don't have the funding to pay for everybody's phone bills and devices”. [P1] **(Q37)** “It would be really neat if apps could have that capacity so you could…you could purchase it as an organization and then be able to give little tickets out to kids so that they could register for the app”. [P6]
	Evidence and safety	**(Q38)** “For us as a service provider, we can't make recommendations on things that are notproven, right? Proven to be effective. There's…we have accountability there”. [P3] **(Q39)** “You know I think if anyone follows sort of the saga of apps and social media there is like, what's a hard delete versus like your account is deactivated? So, all those kinds of concerns around if we're recommending something, do we have a really clear picture that there is safety for clients?” [P4]
–	**Compatibility**	
	Alignment with values and needs	**(Q40)** “So, organizational values for us include sort of a client-centred approach. I think that this app is very client-centred…Another value of ours is continuous quality improvement and that means being really innovative and advancing and doing new things”. [P3] **(Q41)** Well, our overall goal is strengthening our families, communities, and individuals. By this app helping improve one's mental health, that is a part of strengthening overall”. [P1]
	Barriers to compatibility	**(Q42)** “The population that we work with is mostly Indigenous families and children. I feel like, whether it's looking at a holistic approach of the medicine wheel…maybe the app seems very Westernized for the majority of our clients”. [P7]
–	**Engaging service providers**	
	Reminders	**(Q43)** “I think that it is more about education and engagement in other service areas to make sure that they…and like constant reminders I guess that they are also utilizing the tool because I know there are so many other areas in the agency that this would benefit but it's being underutilized in those areas”. [P1] **(Q44)** “We had the presentation, we get weekly emails saying, “don't forget to get people on JoyPop or ask your clients about JoyPop”. That's what got me to reach out to clients”. [P2]
	Training and technical support	**(Q45)** “Like a, do like an education piece. And um…you know, [answering] whatever questions [service providers] have in terms of how to talk to kids about the app and the benefits and all of that stuff”. [P3] **(Q46)** “…keeping it at the forefront and reminding staff, training new hires, or like doing orientations with new hires to make sure everybody has the info”. [P1] **(Q47)** “If someone from our organization was like trained in the app and able to like educate and aid in that way that would be helpful”. [P7]
	Value and rationale	**(Q48**) “I mean, there's the whole change management piece, right? Creating the…that idea that “Why this is good? What's in it for me? What's the value that I'm gonna get out of that?” right, so you have to sort of sell that to the staff who are going to be talking with kids about it”. [P3] **(Q49)** “So, if someone [within the organization] said to me this will work like alongside you to help your client with overall mental health and wellness, that would make me want to be more interested in introducing it to my clients”. [P2]

#### Perceived usefulness

Perceptions of the app's usefulness in improving their clients' mental health and/or enhancing current services were predominantly related to benefits for clients, benefits to services, and barriers preventing the app's usefulness for clients/or services.

##### Benefits to clients

All participants described the general benefits the JoyPop™ app could provide to clients. For example, service providers discussed how the app could benefit youth and underserved populations by increasing access to mental health supports, coping skills, and social support (e.g., Circle of Trust feature; Q1). Three participants additionally discussed how the app could help clients develop healthy habits and autonomy outside of services (Q2 + 3). All participants described specific benefits that the app could provide clients, such as improved coping skills and self-regulation, increased relaxation, improved self-awareness and self-monitoring, and being a helpful distraction when experiencing stress (Q4). Consequently, due to high rates of trauma-related symptoms among the clients they serve, many saw the app as being a beneficial adjunct to treatment for clients to cope with trauma-related difficulties (e.g., avoidance, difficulties with sleep and concentration, negative affect, hyperarousal) (Q5 + 6).

##### Benefits to services

All participants described how the app could benefit *pre-treatment* services, such as supporting clients on the waitlist and decreasing waitlists by preventing a client's need for intensive services or supporting low-risk youth who may not need intensive services (Q7). Many participants also described how the app could help clients build comfort before seeking more intensive services, which was considered necessary because of the high rates of trauma among their clients and the potential dangers of delaying treatments (Q8). All participants described how the app could facilitate and improve outcomes for clients and clinicians during *active treatment*. Many participants discussed how the app could help clients between sessions by facilitating homework and skill reinforcement (Q9). Participants further described how the app could bolster in-person sessions by providing clinicians with more tools to meet clients' diverse needs and by improving clients' ability to track their moods and experiences, which could provide the clinician with more information to help support them (Q10 + 11). Lastly, participants discussed how the app might reduce time spent in counselling and the need for acute care services (e.g., hospitalizations). Five participants discussed how the JoyPop™ app could benefit *post-treatment* services as a discharge recommendation to ease a client's transition out of services and support youth with evidence-based coping skills after counselling (Q12 + 13).

##### Barriers

Every participant discussed useful additions that would improve the app for youth and their services, such as increasing the variety of coping skills and activities, adding notifications and rewards, and integrating different language options (Q14 + 15). Three participants talked about difficulties relating to youth engagement based on their experiences trying to engage youth to try the app throughout the pilot study (Q16). Others discussed the general problems in having youth clients follow treatment recommendations and how this would likely generalize towards the JoyPop™ app if it became a treatment option (Q17). Although these participants highlighted the app's benefits, engaging youth to try the app was mentioned as a significant barrier. Almost all participants commented on the need to ensure the appropriate use of the app by clients and service providers to supplement usual services to minimize risk and harm. These participants were cautious about the potential risks of using the app to replace treatment or with clients relying on an app and forgoing other interventions (Q18).

#### Perceived ease of use

Participants highlighted aspects of the app's design, functions, and features that could either improve or create barriers to the ease of using the app for clients and services.

##### Facilitators

All participants described the ease for clients in their services to learn and use the app based on their initial perceptions of its simple design and layout and because of clients' familiarity and comfort with using technology (Q19 + 20). Six participants discussed the ease for service providers to use the app and integrate it into regular services. Overall, participants did not foresee any serious resistance toward integrating the app. Reasons were associated with the ease with which they integrated mHealth apps in the past (Q21). Others referred to the simplicity of setting up clients with the app for the pilot study (Q22).

##### Barriers

Five participants commented on barriers for clients that would make learning and using the app more challenging. Because of significant socioeconomic barriers, especially for Indigenous clients, most participants talked about how the cost and lack of appropriate devices could make accessing the app challenging for some clients (Q23). Six participants described barriers to integration that may reduce acceptance of the app into usual services. Perceptions centred around workload concerns and service provider feelings (e.g., distrust of apps in general) and characteristics (e.g., personality) towards integrating a mHealth app into services (Q24 + 25).

#### Attitudes towards use

##### Positive feelings

Consistent with their perceptions of the JoyPop™ app's usefulness, each service provider expressed positive feelings toward various client benefits, including improved accessibility, confidential support, and enhanced coping skills in various life domains (Q26 + 27). Two participants highlighted the positive feelings that their client, who participated in the pilot study, had while using the app during active treatment (Q28). All participants expressed positive feelings towards the JoyPop™ app's integration into usual services, often because of its usefulness to improve their services at different stages of treatment and their overall impressions of the app as a tool to help clients (Q29 + 30).

##### Concerns

Six participants highlighted concerns for youth and clients using a mHealth app. Participants were concerned that the JoyPop™ app might confuse clients who do not speak the same language or have lower literacy levels (Q31). Others had concerns over the adverse effects (e.g., avoiding more practical skills, isolation) of youth spending more time on their phones (Q32). Five participants had concerns with integration of the app into usual services. Participants expressed concerns about potential difficulties engaging clients in long-term app use to harness its effectiveness (Q33). Some participants also had concerns about protecting client privacy and the ownership of the information gathered from the app (Q34).

#### Facilitating conditions

Service providers identified various organizational and technological factors that would influence the overall support and integration of the JoyPop™ app into services. Factors included the organizations' accountability in providing quality, ethical, and equitable services, whether the app is consistent with their organization's current values and needs, and their responsibilities in engaging service providers to support the continued and appropriate use of the app.

##### Accountability

All participants described the importance and difficulties for the organization in ensuring clients were provided equitable access to the JoyPop™ app via devices and funding, especially for clients that may face increased inequities and lack the money or devices to use the app (Q35 + 36). However, participants were optimistic about adapting and finding ways that the organization could support clients in getting access to devices and the app. For example, two participants discussed working with schools and developers (e.g., creating a joint purchase plan) to increase client access; some believed their organization could cover client costs (Q37). Four participants highlighted the importance of establishing evidence and safety behind the app for their organizations to ensure clients were provided with a low-risk and effective intervention. A clear picture of the safety and evidence associated with the JoyPop™ app was essential for the organization to support its integration into usual services (Q38 + 39).

##### Compatibility

Participants described how the app aligned with the values and needs of their organization because of the clear connection between their organization's continuous quality improvement, strength-based, and client-centred approaches and the JoyPop™ app (Q40 + 41). Four participants (working with primarily Indigenous clients) described barriers to compatibility. They suggested changes to the app that could increase its compatibility with the values and needs of their Indigenous-health-specific organization. The main barriers to compatibility were the lack of cultural adaptations within the app, which did not align with the organization's fundamental values (Q42).

##### Engaging service providers

Four participants described the importance of ensuring reminders were provided to service providers about utilizing the JoyPop™ app. Reminders were discussed in the form of team meetings, sharing opportunities within organizational departments, and encouragement to staff from managers (Q43). Two participants talked about email reminders they received from their organization about offering clients the opportunity to participate in the pilot study, which increased their engagement with the JoyPop™ app (Q44). Six participants described the importance of having training and technical support with the JoyPop™ app. The training included educating and ensuring new staff members are competent in using the JoyPop™ app with clients (Q45 + 46). Participants also discussed having one member in their organization with experience and training that could provide technical support for others if needed (Q47). Four participants described the significance of having the organization explain the value and rationale of integrating the JoyPop™ app into services, such as a member within the organization (e.g., manager) describing the benefits of the JoyPop™ app in order to engage service providers (Q48 + 49).

## Discussion

National Canadian eHealth guidelines recommend a multi-method approach to develop and evaluate mHealth interventions, including gleaning the perspectives of service providers and users to assess acceptance (5, 20). These perspectives are critical in tailoring apps to the local needs of those engaging in or working within mental health systems and supporting long-term acceptance and uptake of mHealth apps into models of care (20, 24). Therefore, this study aimed to evaluate the acceptance of the JoyPop™ app among a clinical sample of youth and service providers using the TAM, at the two largest mental health agencies in Northwestern Ontario.

### Acceptance of the JoyPop™ app

Overall, the results support the acceptance of the JoyPop™ app among a clinical sample of youth and mental health service providers. Because important feedback/recommendations were identified using theoretical frameworks of technology acceptance, results will help developers ensure that future changes to the app are consistent with local needs and established constructs proven to increase acceptance and continued use (17, 19). Recommendations can also be leveraged to increase the overall utility of the app in future studies with youth and service providers.

#### TAM constructs

The perceived usefulness of the JoyPop™ app to improve mental health and well-being was highlighted by all youth and service providers. Most participants described how the app was useful because it facilitates accessibility to coping skills and mental health support for individuals who face barriers (e.g., geographical distance, long wait times) when accessing services. Each group of participants also reported that the app improved, or could improve, mental health via improved sleep hygiene, self-awareness, self-expression, and emotion regulation. The usefulness of the JoyPop™ app as a novel tool for mental health services in Northwestern Ontario was also evident. All service providers perceived the JoyPop™ app as a valuable addition for their diverse clientele and services because of its potential utility at various stages of treatment (i.e., pre/active/post-treatment). These results are promising because one of the primary purposes of the app is to provide a transdiagnostic, easily accessible intervention for youth and mental health agencies that targets general well-being and coping via improved emotion regulation. Importantly, these results are consistent with research suggesting that the usefulness and benefit (e.g., added value) of using mHealth technology are among the most important factors influencing youth and service providers' acceptance of mHealth apps (17, 19, 22). Although not all youth used every feature available in the JoyPop™ app, each youth identified specific features relevant to their needs. These results support the usefulness of the JoyPop™ app because it provides a range of features that can help meet the various needs of youth and is consistent with research highlighting the importance variety plays in the long-term use and acceptance of mHealth apps (21, 22).

Both youth and service providers perceived the JoyPop™ app as easy to use and/or integrate because of its simple design, aesthetic appearance, and practical functionality. These results are consistent with the broader literature demonstrating increased acceptance and long-term uptake of mHealth apps with simple designs, straightforward functions, and a pleasant appearance (17, 19, 22). Attitudes expressed by youth and service providers also support the acceptance of the JoyPop™ app. Both youth and service providers converged in their positive feelings and attitudes about the usefulness and ease of using the JoyPop™ app. These findings are promising because expressed positive attitudes toward mHealth apps are among the strongest predictors of technology acceptance and long-term engagement (24, 26). These results also align with research suggesting that individuals are more likely to accept and express positive feelings toward efficient and helpful apps with an intuitive, straightforward design (19, 22). One interpretation of these findings is that the positive attitudes expressed by youth and service providers highlight fundamental aspects, designs, and functions within the JoyPop™ app that are essential to its overall acceptance.

Youth and service providers converged on their recommendations to improve the JoyPop™ app. Both discussed the need for specific additions and enhancements of current features that could increase the usefulness and ease of the app for usual services and youth mental health. Additions included ensuring cross-platform compatibility to increase accessibility for clients who do not have iOS devices, adding a built-in notification system, a tracking system (e.g., tracking mood over time), and a larger variety of games to increase usage and engagement. Further, adding a more diverse array of coping skills (e.g., mindfulness meditations) was recommended to support youth's diverse needs better. Enhancements to features were suggested, such as adding more pencil sizes to the Art feature and grid lines to the SquareMoves feature, along with increasing the number of moods in the Rate My Mood feature to increase feature utility (e.g., increased mood monitoring), ease of use, and long-term engagement. Incorporating user feedback is needed because the design, variety, and functionality play a critical role in the long-term acceptance and continued use of mHealth apps (21, 22). Research demonstrates that app users are unlikely to continue using mHealth apps that do not engage them immediately (72). Failing to incorporate app feedback suggested and validated by primary users in the development and evaluation of mHealth apps can also result in developers missing vital technical improvements and set back evaluative research and the overall clinical effectiveness of an app (17, 72). Developers must consider, implement, and evaluate these recommendations to increase acceptance among diverse samples of youth and service providers while providing a more user-friendly and user-centred mHealth app tailored to local needs (5, 20, 72). Developers of the JoyPop™ app have and will continue to consider the perspectives and recommendations from users at each stage of its development and implementation. This process will ensure the app is user-focused, engaging, and effective over long periods.

#### Facilitating conditions

Essential to the comprehensive evaluation in the present study was the addition of the facilitating conditions construct from the UTAUT to capture important organizational factors influencing mHealth acceptance among service providers. All participants found the app compatible with their organization's current values and needs because of its strength-based and client-centred approach. These findings support the acceptance of the JoyPop™ app in broader healthcare contexts because the compatibility of an app with an organization's values and needs is often associated with increased acceptance among service providers (18, 19).

Relevant needs were identified within each organization that could increase or decrease future acceptance of the JoyPop™ app into regular services. For example, because some clients may be unable to afford appropriate devices to use the app, ensuring that the organization could provide clients equal access to the app was a key factor influencing acceptance. These findings are consistent with research showing that lack of proper funding, costs, and reimbursement issues can hinder service providers' acceptance of mHealth apps (19, 73). It will be vital for developers and researchers of the JoyPop™ app to continue working with service providers and their accompanying organizations to find new ways to improve equitable access. For instance, studies have shown that payment models and integrating mHealth tools into healthcare plans can increase acceptance among service providers (5, 19, 20).

The need for a clinical evidence base and adequate training and education to engage service providers were also identified as important organizational needs influencing the acceptance of the JoyPop™ app. These needs are consistent with research highlighting the important influence clinical effectiveness and appropriate training and education have on service providers' acceptance of mHealth apps (18, 19). The results suggest that continuing to evaluate the JoyPop™ app's clinical effectiveness is important to establish its evidence base and promote acceptance, trust, and quality interventions among service providers (18, 19). Also, continuous support from developers in terms of guidance and the provision of education to service providers is an important first step to maximize acceptance and continued use if the app is integrated into usual care (18, 19). Implementation science will be critical in achieving optimal use, acceptance and long-term adoption of the app among mental health organizations. For example, identifying “champions” (dedicated individuals supportive of the app) within organizations may bolster the promotion, proper use, and eventual acceptance of the JoyPop™ app in complex healthcare settings (74).

### The JoyPop™ app and coping with adversity and trauma

All youth, including the three Indigenous youth, expressed how the app was useful in improving mental health and emotion regulation. One participant specifically noted that using the app helped address emotion regulation difficulties associated with their past trauma (e.g., they used Rate My Mood to document heightened emotional arousal). These findings are important given that many youth presenting for mental health services in Northwestern Ontario do so with difficulties related to emotion regulation. This is also relevant as Indigenous youth within this region experience particular difficulties with adjusting to past trauma (49). It is well established that neurocognitive and neurobiological changes occur throughout development in response to childhood trauma and adversity (51, 52). These changes influence the functioning of neurobiological systems associated with emotional arousal and emotion regulation and leave children with response propensities that affect how they respond to stress (75, 76). Developing certain response styles (e.g., hypervigilance, avoidance) related to early trauma and adversity may provide some protective benefits within the original context (e.g., recognizing and attempting to avoid contact with the threat), however, they may be a liability in other context in which they are not required (e.g., at school) and influence future resilience (75, 77).

In addition, many service providers in the present study were willing and eager to integrate the JoyPop™ app into usual care because of its potential utility in improving trauma-related symptoms at various stages of treatment. These perspectives align with research demonstrating the utility of mHealth apps in treating trauma, especially when used as an adjunct treatment (10, 14). The results of the present study (e.g., self-reported improved emotion regulation) and evidence that the JoyPop™ app confers the greatest benefits on emotion regulation for those who have experienced more childhood adversity (29), suggest that the app may be able to bolster an individual's capacity to manage the impact of trauma by facilitating resilience through adaptive coping. The results are consistent with research showing that mHealth apps can be a helpful standalone intervention for young people (11) and that resilience can be harnessed and increased through interventions that target emotion regulation (78), training programmes [e.g., mixed interventions, mindfulness; (79)], and strength-based primary prevention efforts (80). Future research specifically testing these speculations is required.

### Limitations and future directions

There are some significant limitations concerning the results of this study. First is that all participants were female. Research suggests there are sex and gender differences in the acceptance of new technology for youth and service providers (81, 82); therefore, future research with other gender identities (including boys and men) is needed. A second limitation is that out of 20 youth who were invited to participate in the qualitative study, only six completed an interview and 1 youth (representing 14% of the initial sample) left the study because of discomfort. It is also likely that many service providers were aware of their opportunity to participate in the study but chose not to. This may result in a non-response sample bias, influencing the results and reducing the representativeness of the sample and generalizability of the results (83). For example, participants in the pilot study who felt more positively about the app and/or interacted with it more often may have been more likely to participate in this qualitative study. Further, important perspectives of youth who may be less comfortable with discussing their experiences during an interview may have been missed. However, upon comparison, youth who completed the qualitative study and youth that were invited but did not complete it were similar in age, sex, and ethnicity. Youth ratings of satisfaction and acceptance were also similar. These results provide some reassurance that the sample of youth who participated in the qualitative study were similar to those that participated in the pilot study but chose not to participate in the qualitative study.

Another limitation is that only those that completed the pilot study were eligible to participate in this qualitative study. Also, no demographic data were available for service providers who did not participate in the study. Assessing acceptance among youth who did not complete the pilot study and service providers who did not participate may have resulted in more negative feedback. Negative feedback is essential for informing continued adaptation and tailoring of the app to meet the diverse needs of youth and healthcare organizations (17, 19). Research has shown that there are certain factors (e.g., socioeconomic status, age, mental health diagnoses, personality traits, culture) that influence youth and service providers' perspectives on mHealth app acceptance either negatively or positively (17, 19, 84). Future research should gather more information about these factors to better understand youth and service providers' acceptance of the JoyPop™ app. Usage of the app can also influence acceptance of mHealth apps and unfortunately we were unable to obtain and make use of objective usage data from the app (i.e., details about how often and how long each feature was used). Gathering and examining data on overall app usage including patterns and duration of feature usage would provide important insight into the characteristics of youth who use each feature and acceptance of each feature to better serve the diverse needs of youth. Future research should also consider strategies to explicitly seek out more critical feedback (e.g., contact youth who dropped out of pilot study early, as they may be more likely to hold more negative views). It may also be beneficial to seek out a diverse group of participants to watch a video or test out the app for a shorter period of time (e.g., a few days) and then ask them about their experiences.

A third limitation is related to the broad age range of the youth sample (12–17 years). Adolescents is marked by significant changes in biological (e.g., puberty), psychological (e.g., emotion regulation), behavioural (e.g., risk behaviours) and social development [e.g., changes in school environment; (85)] and age has been shown to moderate app acceptance (17, 84). Examining larger samples of youth from early adolescents to later adolescence may reveal specific positive and negative feedback on the acceptance of the JoyPop™ app. For example, youth in the upper ranges (17–18) may be more likely to express themselves verbally (e.g., using the Journal feature) vs. youth in the lower ranges (12–13), who may be more visual (e.g., using the Art feature). However, it is important to note that the JoyPop™ app purposefully has various features to capture these potential differences and meet the needs of all ages of youth. However, future research would still benefit from understanding app acceptance among older and young youth.

A final limitation is that we only had one coder which could impact the interpretations derived from the data (86). Though having more than one coder to examine the inter-coder reliability can help validate the data analysis, more important is having multiple researchers evaluate the content of codes to highlight disagreements and provide insight to refine the coding process (86, 87). In addition, having detailed notes of all decisions made throughout the coding process and the organization of the data into categories is essential to facilitate the consistency and trustworthiness of coding procedures if only one coder is possible (87). We reduced this limitation by having the first, second, and last authors meet throughout each step of data analysis to highlight disagreements and reach a consensus. The first author also kept detailed memos and notes of all their decisions in the form of an audit trail.

## Conclusion

This qualitative study guided by the TAM provides insight into the factors that influence acceptance of the JoyPop™ app among youth and service providers in Northwestern Ontario. All youth and service providers found the app useful and easy to use and expressed positive attitudes toward using the app, along with recommendations to improve acceptance in future iterations. Service providers also acknowledged and highlighted important organizational factors that may influence acceptance of the JoyPop™ app into usual care. The present research contributes to a growing body of evidence supporting the JoyPop™ app as an accessible, helpful, and timely intervention to reduce barriers to mental health services and support diverse youth in Canada, especially in underserved areas like Northwestern Ontario. The recommendations and suggestions derived from the results are essential in ensuring that future iterations of the JoyPop™ app meet the needs of youth and service providers.

## Data Availability

The original contributions presented in the study are included in the article/Supplementary Materials, further inquiries can be directed to the corresponding author.

## Footnotes

^1^A supplementary analysis was conducted to examine app acceptance on a quantitative measure used in the pilot study in an effort to compare youth that completed an interview in the qualitative study vs. those that chose not to participate or did not complete the interview in the qualitative study. This comparison is mentioned at the end of the results section.
