# Supplementary material for: Female youth and mental health service providers' perspectives on the JoyPop™ app: a qualitative study
Source: Front Digit Health. 2023 Sep 27;5:1197362. doi: 10.3389/fdgth.2023.1197362 (PMC10566298; doi:10.3389/fdgth.2023.1197362)
Supplement: Supplementary file 1 [file Table1.docx]

Supplementary Material

Qualitative Evaluation of Youth and Mental Health Service Provider Acceptance of the JoyPop^TM^ App

Ishaq Malik, Arnaldo Perez, Elaine Toombs, Fred Schmidt, Janine Olthuis, Jaidyn Charlton, Crystal Squier, Kristine Stasiuk, Tina Bobinski, Aislin R. Mushquash^*^

*** Correspondence:** Dr. Aislin Mushquash aislin.mushquash@lakeheadu.ca

**Supplementary File S1: Youth and Service Provider Interview Guides**

Youth Interview Guide

**Probes:** Identified by letter and can be omitted if participants answer the questions in their answer to the broader question.

**Introduction:** We are interested in your experience and satisfaction with the app. We will ask you a series of questions but please feel free to provide any additional details based on your experiences over the last 4 weeks.

1. Approximately how often did you use the app?
2. Have you ever used anything like this before? If yes, please describe.
3. What is your understanding of the purpose of the app?
   1. How did using the app affect you?

b. How did using this app affect the quality of your life?

c. How did using the app affect your mental health, if at all? *Prompt -* Describe.

d. In what ways could this app be a useful addition to your usual mental health services? *Prompt -* Why or Why not?

1. Did you find the app easy to learn and use? If yes/no, please describe.
   1. Did anything about the app make it difficult to learn and use? If yes, please describe.
   2. Did anything about the design (e.g., layout, appearance) of the app make it easy or difficult to use? Please describe.
2. Overall, please tell me about your experience using the app.
   1. How did you feel when using an app like this? Why?
   2. How did you feel when you first started using it? Why?
   3. How did you feel about using it on an ongoing basis? Why?
   4. How would you feel about using this app in the future? Why?
3. Which features did you use the most often? Why?
4. Which features did you use least often? Why?
5. Which features did you find most useful? Why?
6. Which features did you like the most? Why?
7. Which features did you find easiest to use? Why?
8. What features did you find most difficult to use? Why?
9. What recommendations or suggestions do you have to improve the app?
   1. What you make the app easier/less effort for you to use? Why?
   2. What would make you more likely to use the app?
   3. What would make the app more useful to you? Why?
   4. What would make this app a more positive experience for you? Why?
10. Anything else you would like to share about your experience with the app?

Service Provider Interview Guide

**Introduction:** We are interested in your experience integrating the App into usual mental health services. We will ask you a series of questions but please feel free to provide any additional details based on your experiences over the last 4 weeks.

1. What is your position at your place of work?
2. What services do you provide in your role at work?
3. What age of clients do you work with?
4. Roughly how many years of experience do you have working in your field?
5. What is your sex?
6. What is your ethnicity?
7. What is your age?
8. Have you ever used anything like this before with clients? If yes, please describe.
9. What is your understanding of the purpose of the app?
   1. In what ways could this app be useful for clients? Why or why not?
   2. How could this app affect your job? Why or Why not?
10. What were (or are) your expectations about what the app would/could do for youth?
    1. How did (or how could) the app meet your expectations?
    2. How do you think youth feel about using an app like this? Do you think youth have found it or would find it easy to use?
    3. How do you feel about youth using an app like this? Why?
11. Overall, please tell me about your experience (or thoughts about) integrating the app into the mental health services you typically provide.
    1. How do you feel about integrating an app like this into usual care? Why?
    2. What is your impression of the usefulness of the app as an adjunct to usual services for youth in and/or seeking mental health care?
    3. Did you find it easy to integrate (or do you think it would be easy to integrate)? Why or why not?
    4. What would make it better/easier to integrate the app into usual services?
12. What factors in your organization (or organizational environment) would make integrating this app into usual care easier or more difficult? Why?
    1. Do you think this app is compatible (e.g., consistent with your current values and needs) with your current position? Why or why not?
    2. What support from your organization would be helpful to you if you were to want to integrate this app into usual services? Why?
13. What recommendations or suggestions would you make to facilitate staff to use the app as an adjunct to services?
    1. What would make it more useful for you or your services? Why?
    2. What could your organization do to support the integration of this app into usual care? Why?
    3. In what ways could this app ne received more positively by staff? Why?
14. What recommendations or suggestions do you have to increase the likelihood of youth using the app?
    1. What do you think would make this app more useful to youth?
    2. What do you think would make this app easier for youth to use?
    3. What do you think would make this app a more positive experience for youth?
15. Anything else you would like to share about your experience with the app?

**Supplementary Table S2: COREQ Checklist**

COREQ: Consolidated Criteria for Reporting Qualitative research: A 32-item Checklist for Interviews and Focus Groups^36^

Note: IM: Ishaq Malik, AP: Arnaldo Perez , ET: Elaine Toombs, FS: Fred Schmidt, JO: Janine Olthuis, JC: Jaidyn Charlton, CS: Crystal Squier, KS: Kristine Stasiuk, TB: Tina Bobinski, AM: Aislin Mushquash. TAM: Technology Acceptance Model, TAM+: Extended Technology Acceptance Model.

| **Section/Topic** | **Item No** | **Checklist item** | **Information** |
| --- | --- | --- | --- |
| **Domain 1: Research team and reﬂexivity** | | | |
| Personal Characteristics | | | |
| *Interviewer/facilitator* | 1 | Which author/s conducted the interview or focus group? Interviewer/facilitator | IM. |
| *Credentials* | 2 | What were the researcher’s credentials? E.g. PhD, MD | - IM: HBA  - AP: PhD  - ET: PhD  - FS: PhD  - JO: PhD  - JC: HBA  - CS: MSW  - KS: HBSW  - TB: MSW  - AM: PhD |
| *Occupation* | 3 | What was their occupation at the time of the study? | - IM is a Masters student in the Clinical Psychology program at Lakehead University.  - ET is a Postdoctoral Researcher at Lakehead University and clinical psychologist at Dilico Anishinabek Family Care.  - AP is the Assistant Clinical Professor in the Faculty of Medicine and Dentistry at the University of Alberta.  - FS is the Director of Continuous Quality Improvement and a clinical psychologist at Children’s Centre Thunder Bay.  - JO is an Associate Professor at the University of New Brunswick.  -JC is a Masters student in the Clinical Psychology program at Lakehead University.  - CS is the manager of Clinical and Counselling Services at Dilico Anishinabek Family Care.  - KS is the Assistant Director of Mental Health and Additions at Dilico Anishinabek Family Care.  - TB is the Director of Mental Health and Addictions at Dilico Anishinabek Family Care.  - AM is an Associate Professor in the Department of Psychology at Lakehead University and a clinical psychologist. |
| *Gender* | 4 | Was the researcher male or female? | - IM (male)  - AP (male)  - ET (female)  - FS (male)  - JO (female)  - JC (female)  - CS (female)  - KS (female)  - TB (female)  - AM (female) |
| *Experience and training* | 5 | What experience or training did the researcher have? Relationship with participants | - IM has experience in clinical interviewing and was a mental health counsellor at an Indigenous organization for the past three years.  - AM and AP, both have extensive training in clinical and research interviewing.  - All other authors not involved in the data analysis. |
| Relationship with participants | | | |
| *Relationship established* | 6 | Was a relationship established prior to study commencement? | No relationships established prior. |
| *Participant knowledge of the interviewer* | 7 | What did the participants know about the researcher? e.g. personal goals, reasons for doing the research | Participants knew the rationale and purpose of the interviews. A detailed information letter detailing the purpose, design, and goals of the study was provided and explained by the researcher. |
| *Interviewer characteristics* | 8 | What characteristics were reported about the interviewer/facilitator? e.g. Bias, assumptions, reasons and interests in the research topic | The multidisciplinary team involved in this study promoted a wide variety of interpretations but inevitably brought various expectations and assumptions to the data collection and analysis process. For example, there may be an attachment towards the JoyPop app to not only demonstrate its effectiveness, but to be a useful addition to usual services at both organizations. There also may be pressure on participants to view the app positively because individuals with influence at each organization are part of the research team.  Several steps were taken to minimize potential bias (also see Table 2). Each participant was clearly informed that their participation or non-participation would not affect their access to services or employment at their respective organizations. CS, KS, TB, ET, JO, and FS were only involved in study promotion and were not part of the data collection and analysis processes. JC was only involved in data collection. Questions in the interview guides were neutrally and openly worded to avoid signs of the researchers’ opinions. IM also kept a reflexive journal that was used before and after interviews. This allowed him the opportunity to identify any anticipated barriers associated with power and privilege between the researchers and the participants while allowing him to identify, and reduce, any biases or challenges he had |
| **Domain 2: study design** | | | |
| Theoretical framework | | | |
| *Methodological orientation and*  *Theory* | 9 | What methodological orientation was stated to underpin the study? e.g. grounded theory, discourse analysis, ethnography, phenomenology, content analysis | Qualitative descriptive design. Deductive and inductive content analysis. |
| Participant selection | | | |
| *Sampling* | 10 | How were participants selected? e.g. purposive, convenience, consecutive, snowball | Youth: purposive/  Service Providers: purposive and snowball. |
| *Method of approach* | 11 | How were participants approached? e.g. face-to-face, telephone, mail, email | All but one participant approached via email. Two youth were approached in-person when receiving compensation for the larger evaluation. |
| *Sample size* | 12 | How many participants were in the study? | Final sample size (*N =* 13): Youth (*n* = 6), Service Providers (*n* = 7). |
| *Non-participation* | 13 | How many people refused to participate or dropped out? Reasons? | One. Youth decided they no longer wanted to continue after the first question of the interview. |
| *Setting of data collection* | 14 | Where was the data collected? e.g. home, clinic, workplace | Zoom (*N* = 10): Four youth from their home, six service providers from their place of employment.  Phone (*N = 2)*: Youth from their home, Service Provider from their own office. In-person (*N = 1):* Youth interview conducted at location they were receiving mental health services. |
| *Presence of non-participants* | 15 | Was anyone else present besides the participants and researchers? | Caregiver present for one in-person youth interview. |
| *Description of sample* | 16 | What are the important characteristics of the sample? e.g. demographic data, date | See **Participant Demographics** in manuscript. |
| Data collection | | | |
| *Interview guide* | 17 | Were questions, prompts, guides provided by the authors? Was it pilot tested? | Yes. See **Supplementary File S1.** Youth and Service provider interview guides piloted with two youth and two service providers before being applied to entire sample. |
| *Repeat interviews* | 18 | Were repeat interviews carried out? If yes, how many? | No. This was to reduce participant burden, scheduling issues. |
| *Audio/visual recording* | 19 | Did the research use audio or visual recording to collect the data? | Audio. |
| *Field notes* | 20 | Were ﬁeld notes made during and/or after the interview or focus group? | Yes, IM kept a reflexive journal that he used before and after interviews to identify biases, challenges, assumptions while recording and reflecting on important details after each interview. |
| *Duration* | 21 | What was the duration of the interviews or focus group? | See **Participant Demographics** in manuscript**.** |
| *Data saturation* | 22 | Was data saturation discussed? | N/A. Recruitment not based on data saturation. |
| *Transcripts returned* | 23 | Were transcripts returned to participants for comment and/or correction? | No. This was to reduce participant burden, scheduling issues. |
| Domain 3: analysis and ﬁndingsz  Data analysis | | | |
| *Number of data coders* | 24 | How many data coders coded the data? | One. Each stage of the data analysis process was discussed and reviewed to reach consensus by IM, AM, and AP. |
| *Description of the coding tree* | 25 | Did authors provide a description of the coding tree? | Yes. See **Figures 2 and 3 and Table 2 and 3.** |
| *Derivation of themes* | 26 | Were themes identiﬁed in advance or derived from the data? | Both. TAM and TAM+ constructs were identified as major categories in advance. General and subcategories within major categories were derived from data. Data that did not fit into pre-determined main categories was inductively coded (derived from data) to form categories. |
| *Software* | 27 | What software, if applicable, was used to manage the data? | NVivo Software 12 |
| *Participant checking* | 28 | Did participants provide feedback on the ﬁndings? | No. This was to reduce participant burden, scheduling issues. |
| Reporting | | | |
| *Quotations presented* | 29 | Were participant quotations presented to illustrate the themes / ﬁndings? Was each quotation identiﬁed? e.g. participant number | Yes. See **Results** in manuscript. |
| *Data and ﬁndings consistent* | 30 | Was there consistency between the data presented and the ﬁndings? | Yes. See **Results** in manuscript **and Figures 2 and 3 and Table 2 and 3.** |
| *Clarity of major themes* | 31 | Were major themes clearly presented in the ﬁndings? | Yes. See **Results** in manuscript **and Figures 2 and 3 and Table 2 and 3.** |
| *Clarity of minor themes* | 32 | Is there a description of diverse cases or discussion of minor themes? | Yes. See **Results** in manuscript **and Figures 2 and 3 and Table 2 and 3.** |
